# The Importance of Early Identification of Alpha-1 Antitrypsin Deficiency

**DOI:** 10.7759/cureus.3494

**Published:** 2018-10-25

**Authors:** Barjinder S Buttar, Mark Bernstein

**Affiliations:** 1 Internal Medicine, Zucker School of Medicine / Northwell Health Mather Hospital, Port Jefferson, USA

**Keywords:** aatd, emphysema, copd, cirrhosis, prolastin, screening, alpha-1 antitrypsin deficiency

## Abstract

Alpha-1 antitrypsin deficiency (AATD) is a common genetic disorder that is easily managed if diagnosed and treated at an early age. It is often missed, however, especially in patients with long histories of smoking and alcohol use. This is mainly due to a lack of awareness and proper screening of the disorder, especially in the primary care setting. Here, we will focus on a case report of a young male whose diagnosis and treatment of AATD was significantly delayed. His lung and liver complications had initially been attributed to his smoking and drinking history. This delay could have been avoided by increasing awareness of AATD and through the implementation of novel screening tests that can quickly rule out the disorder in patients presenting with lung and liver disease.

## Introduction

Alpha-1 antitrypsin deficiency (AATD) is a common autosomal recessive disorder. Alpha-1 antitrypsin (AAT) is defined as a protease inhibitor which is encoded by the SERPINA1 gene. M refers to the normal allele while Z refers to the mutated allele. The mutated Z allele is carried by approximately 2 - 3% of the Caucasian population in the United States. Homozygosity of the Z allele, PI*ZZ, is the most common mutation that leads to AATD [1]. Emphysema occurs in these patients as a result of an imbalance between neutrophil elastase and AAT. Neutrophil elastase destroys elastin which is needed for the lung to maintain its elasticity and resilience. AAT acts as an elastase inhibitor which protects the lung from proteolytic degradation of elastin [1]. If AAT is absent or non-functional, the lung no longer has protection from the activity of neutrophil elastase. When viewing a computed tomography (CT) scan of the chest of a patient with AATD, one can appreciate the classic presentation of diffuse emphysema localized to the lung bases [2]. 

Liver cirrhosis occurs from the accumulation within hepatocytes of unsecreted and defective AAT protein. A liver biopsy photomicrograph of a patient with AATD will display the classic periodic acid-Schiff-positive diastase-resistant globules in hepatocyte cytoplasm consistent with retained AAT-Z molecules [3].

The diagnosis of severe deficiency is confirmed with an AAT serum level below the protective threshold of 57 mg/dL. The normal plasma concentration of AAT ranges from 80 to 220 mg/dL [4]. When treating these patients, the goal is to bring AAT levels back up to the normal range which will slow down the progression of emphysema. Currently, there are four pooled human plasma AAT products available to the public: Aralast, Prolastin, Zemaira, and Glassia. All four products are different variations of the same alpha-1 proteinase inhibitor. When administering them, the only United States Food and Drug Administration-approved regimen is 60 mg/kg of body weight, given as weekly infusions [4]. Studies have shown that weekly infusions of human pooled AAT at a dose of 60 mg/kg maintain AAT levels in plasma and epithelial lining fluid above the protective threshold [4].

## Case presentation

**Figure 1:**
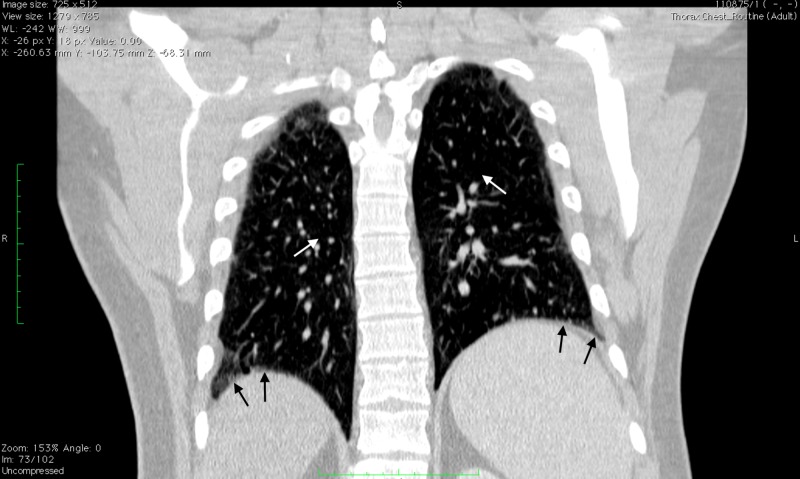
Multidetector Helical Computed Tomography Scan of the Chest, Coronal View This image displays a coronal view of our patient with stable emphysematous changes bilaterally shown by the white arrows and bilateral lower lobe dependent atelectasis shown by the black arrows.

**Figure 2:**
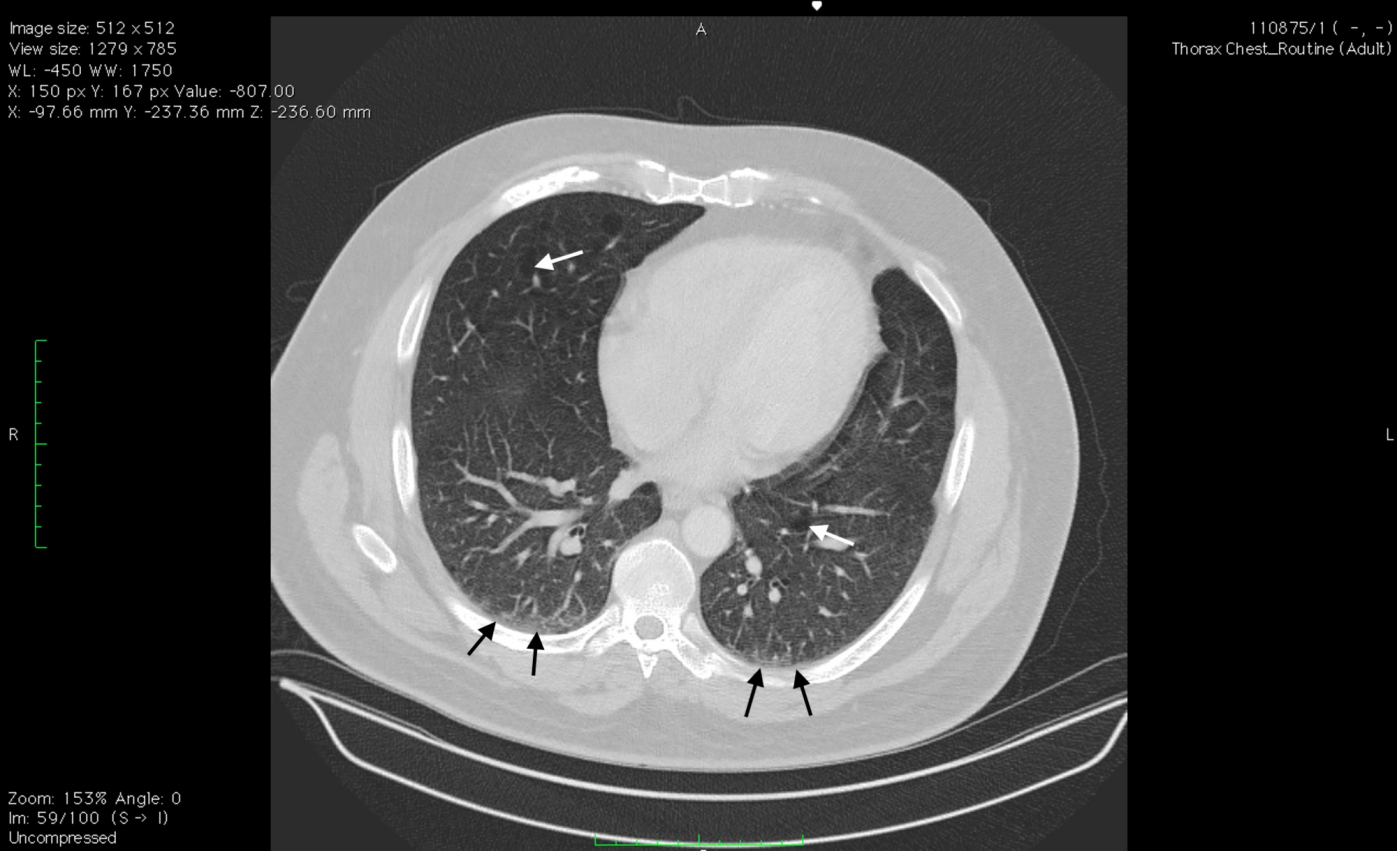
Multidetector Helical Computed Tomography Scan of the Chest, Transverse View This image displays a transverse view of our patient with stable bilateral emphysematous changes shown by the white arrows, as well as bilateral lower lobe dependent atelectasis shown by the black arrows.

This case report will focus on a 39-year-old male with a smoking history of one pack per day and regular alcohol intake of beer for over 20 years. He has had ongoing respiratory and abdominal symptoms which had been attributed to his smoking and drinking history. Starting in his early twenties, he began to have multiple episodes of mild upper respiratory tract infections and bronchitis on a yearly basis. He also experienced mild abdominal discomfort and nausea which would come and go sporadically. High-resolution computed tomography of the chest confirmed emphysematous changes of the lung as shown in Figures 1-2 below.

His liver enzymes had been slowly trending up throughout the years. Over the span of a few months, his aspartate transaminase (AST) level increased from 52 units per liter (U/L) to 58 U/L. His alanine transaminase (ALT) level increased from 81 U/L to 86 U/L. The upper limit of normal for both AST and ALT levels fall in the low 40s U/L. Other markers of liver damage, including serum prothrombin concentrations and serum albumin, were not affected. 

His primary care physician instructed him to quit smoking and drinking, eat healthily, and exercise regularly. He was successful in making these lifestyle changes, but over the next few years, his liver enzymes remained elevated and his lungs continued to show persistent emphysema. There was little to no improvement in his overall symptoms. Due to these abnormal findings, he was tested for alpha-1 antitrypsin deficiency (AATD) and was found to be homozygous for the Z allele (PI*ZZ). His alpha-1 antitrypsin (AAT) level was 18, which is well below the protective threshold of 57. Once the diagnosis was confirmed, the patient was immediately started on weekly Prolastin infusions. Since his diagnosis, our patient has completed eight infusions of Prolastin and will continue to receive weekly infusions for as long as he is able to tolerate them in order to maintain normal concentrations of AAT. His AAT level continues to increase and is now closer to the protective threshold; his liver function tests have improved as well.

## Discussion

Identifying alpha-1 antitrypsin (AATD) in patients at an early age and beginning treatment as soon as possible is important in preventing ongoing damage to both the liver and lungs. Our patient displayed classic signs of bilateral emphysematous changes typical of patients with AATD (Figures 1-2). He also displayed signs of possible liver damage with symptoms of abdominal discomfort and nausea. On routine labs, his liver function tests indicated transaminitis with his liver enzymes slowly trending up throughout the years. Other markers of liver damage, however, remained within normal limits. Due to the mild and sporadic nature of the symptoms, our patient did not undergo a liver ultrasound or biopsy.

**Figure 3:**
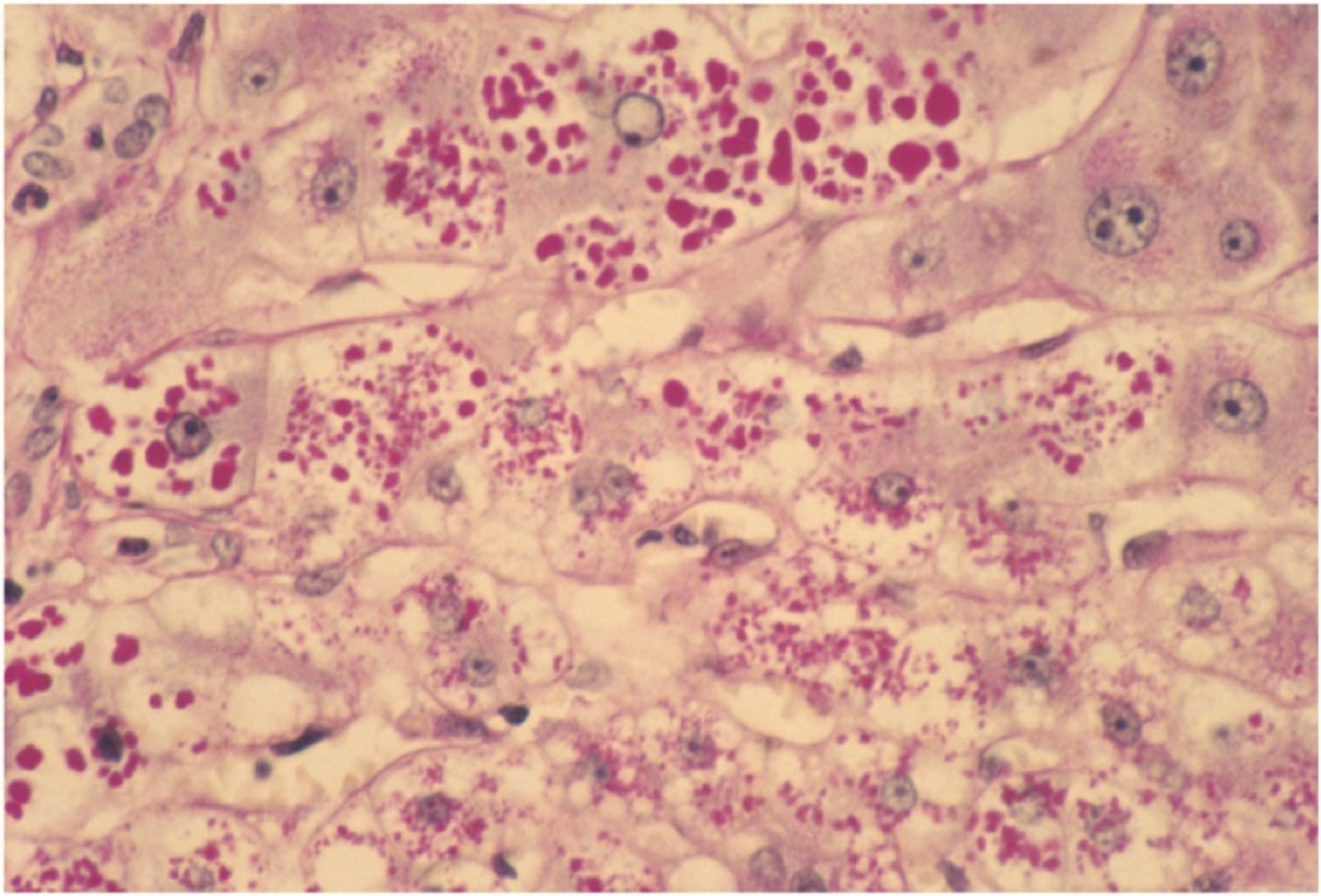
Liver Biopsy Photomicrograph Liver biopsy of a 31-year-old male with alpha-1 antitrypsin deficiency (AATD) showing periodic acid-Schiff-positive diastase-resistant globules in hepatocyte cytoplasm consistent with retained alpha-1 antitrypsin (AAT) molecules, which are identified by the magenta-colored globules [3].

Figure 3 below displays a liver biopsy of a young male diagnosed with AATD. The biopsy shows classic, defective alpha-1 antitrypsin (AAT) molecules accumulating in hepatocyte cytoplasm identified by the magenta-colored globules [3].

Although it is recommended that every chronic obstructive pulmonary disease (COPD) patient be tested for AATD, the condition still remains severely underdiagnosed with a delay of several years between the initial appearance of symptoms and when the diagnosis is actually made [5]. If this patient had been screened for AATD at an earlier age, he would have been started on the appropriate treatment immediately after being diagnosed, minimizing his symptoms. Screening patients for AATD is especially important in the primary care setting because primary care physicians are most likely to be the first to encounter symptomatic individuals [5]. Novel screening tests, such as the AlphaKit® QuickScreen (Grifols International, SA, Barcelona, Spain), which can detect the abnormal AAT protein in capillary whole blood, are great tools that can be used to simply exclude AATD in the overall COPD population [6]. Greulich et al. performed a prospective, real world, diagnostic study on this screening tool to assess its ability to effectively detect AATD in test-naïve COPD patients. The patients were recruited from centers ranging from primary care to tertiary care in Spain and Germany. To evaluate the performance of the test, sensitivity, specificity, positive predictive value, and negative predictive value were calculated and compared to the gold standard genotyping test. The results showed that the AlphaKit® QuickScreen test is an effective tool that can be used to exclude AATD in the primary care setting and in the overall COPD population.

## Conclusions

Alpha-1 antitrypsin disease (AATD) is an under-recognized disease that is estimated to affect 1 - 2% of all chronic obstructive pulmonary disease (COPD) cases. Unfortunately, less than 10% of symptomatic individuals have been properly diagnosed due to a lack of awareness and proper screening of the disorder, especially in the primary care setting. By increasing awareness among physicians and stressing the importance of using AATD screening tests (such as the AlphaKit® QuickScreen), we can do a much better job of diagnosing and treating these patients.
